# CD4^+^CD25^−^Nrp1^+^ T Cells Synergize with Rapamycin to Prevent Murine Cardiac Allorejection in Immunocompetent Recipients

**DOI:** 10.1371/journal.pone.0061151

**Published:** 2013-04-05

**Authors:** Qing Yuan, Shanjuan Hong, Bingyi Shi, Jesper Kers, Zhouli Li, Xiangke Pei, Liang Xu, Xing Wei, Ming Cai

**Affiliations:** 1 Organ Transplant Center, Organ Transplant Institution of PLA, The 309th Hospital of PLA, Beijing, China; 2 National Key Laboratory of Medical Immunology, Second Military Medical University, Shanghai, China; 3 Department of Pathology, Academic Medical Center, University of Amsterdam, Amsterdam, The Netherlands; 4 Organ Transplant Center, The 281th Hospital of PLA, Qinhuangdao, Hebei, China; University of Southern California, United States of America

## Abstract

Besides CD4^+^CD25^+^Foxp3^+^ regulatory T cells (Tregs), other immunosuppressive T cells also participated in the regulation of immune tolerance. Reportedly, neuropilin-1 (Nrp1) might be one of the molecules by which regulatory cells exert their suppressive effects. Indeed, CD4^+^CD25^−^Nrp1^+^ T cells exhibit potent suppressive function in autoimmune inflammatory responses. Here we investigated the specific role of CD4^+^CD25^−^Nrp1^+^ T cells in the setting of the transplant immune response. Through MLR assays, we found that CD4^+^CD25^−^Nrp1^+^ T cells suppressed the proliferation of naive CD4^+^CD25^−^ T cells activated by allogeneic antigen-stimulation. Adoptive transfer of CD4^+^CD25^−^Nrp1^+^ T cells synergized with rapamycin to induce long-term graft survival in fully MHC-mismatched murine heart transplantation, which was associated with decreased IFN-γ, IL-17 and increased IL-10, TGF-β, Foxp3 and Nrp1 expression in the grafts. Importantly, our data indicated that CD4^+^CD25^−^Nrp1^+^ T cell transfer augments the accumulation of Tregs in the recipient, and creates conditions that favored induction of hyporesponsiveness of the T effector cells. In conclusion, this translational study indicates the possible therapeutic potential of CD4^+^CD25^−^Nrp1^+^ T cells in preventing allorejection. CD4^+^Nrp1^+^ T cells might therefore be used in bulk as a population of immunosuppressive cells with more beneficial properties concerning *ex vivo* isolation as compared to Foxp3^+^ Tregs.

## Introduction

Induction and maintenance of donor-specific transplant tolerance is the Holy Grail of organ transplantation that would obviate allorejection and patients' dependency on life-long immunosuppressive treatment [1]. Suppressive cell based therapies have proved to be efficient in promoting tolerance in experimental models [2], [3]. Among them, CD4^+^CD25^+^Foxp3^+^ regulatory T (Treg) cells have drawn considerable attention. Adoptively transferred naturally occurring Tregs have been demonstrated to promote MHC-incompatible organ graft survival in immunologically impaired host rodents, including irradiated [4], Rag2-deficient [5], and T cell-depleted animals [6]. Furthermore, it has been established that the combined treatment of Tregs and a short course of rapamycin is capable to prolong cardiac allograft survival in immunocompetent recipients [7]. However, the population of immunosuppressive T cell is phenotypically and functionally heterogeneous. Increasing evidence indicated that non-Treg immunosuppressive T cells could also be found among CD4^+^CD25^−^ cells [8], [9], as well as TCRαβ^+^CD3^+^CD4^−^CD8^−^NK1.1^−^ (double negative) T cells [10].

Neuropilin-1 (Nrp1), a multifunctional type 1 transmembrane protein involved in axonal guidance as a receptor for semaphorin-3A [11] and in angiogenesis through interactions with vascular endothelial growth factor [12], has been reported as a potent surface marker for murine CD4^+^CD25^+^ Treg cells [13]. Consistently, we observed in previous study that freshly isolated CD3^+^Nrp1^+^ T cells possessed almost to the same extent the ability to suppress proliferation of anti-CD3/anti-CD28 stimulated syngeneic T cell and that they might be even more capable of preventing rejection in a murine skin transplant model as compared with CD4^+^CD25^+^ cells [14].Even though significantly lower as compared to CD4^+^CD25^+^ cells, stable expression of Nrp1 on CD4^+^CD25^−^ cells has been identified in our laboratory as well as others' [15], [16]. Using a mouse model of experimental autoimmune encephalomyelitis, Solomon et al. [16] reported that CD4^+^Nrp1^+^ T cells suppressed effector cell proliferation more efficiently than CD4^+^CD25^+^ T cells, and CD4^+^CD25^−^Nrp1^+^ T cells exhibited similar suppressive function as CD4^+^CD25^+^Nrp1^+^ T cells in preventing disease progression. However, there are still few reports addressing the role of CD4^+^CD25^−^Nrp1^+^ T cells in the transplant immune response. We hypothesize that CD4^+^CD25^−^Nrp1^+^ T cells might have a protective function against allorejection, and therefore we designed this study to test this hypotheses both *in vitro* and *in vivo*.

Here, we show that freshly isolated CD4^+^CD25^−^Nrp1^+^ T cells have efficient suppressive function in the response to the priming by BALB/c (donor) splenocytes *in vitro*. Using a fully MHC-mismatched murine heterotopic cardiac transplant model, we showed that CD4^+^CD25^−^Nrp1^+^T cells synergized with Rapamycin to prevent cardiac allograft rejection, in which CD4^+^CD25^−^Nrp1^+^T cells augment CD4^+^Foxp3^+^ Treg accumulation and induced hyporesponsiveness of the T effector cells in transplant recipients.

## Materials and Methods

### Mice and ethics statement

BALB/c (H2^d^), and C57BL/6 (H2^b^) mice (6–8 wk, weight 20–25 g) were obtained from Joint Ventures Sipper BK Experimental Animal Company (Shanghai, China). All animal experiments were performed in accordance with the National Institutes of Health Guide for the Care and Use of Laboratory Animals, with the approval of the Scientific Investigation Board of Second Military Medical University (Shanghai, China).

### Reagents

Rapamycin was obtained from Sigma-Aldrich. A Mouse CD4^+^ T cell Isolation Kit, CD4^+^CD25^+^ Regulatory T Cell Isolation Kit and goat anti-rabbit IgG microbeads were purchased from Miltenyi Biotec Company (Aubum, CA). Anti-Nrp1 antibody was provided by the Abcam Company (Cambridge, MA). Fluorescent dye-conjugated monoclonal antibodies that specifically recognize CD4, Foxp3 and isotype IgG were purchased from BioLegend Company (San Diego, CA) and eBioscience (San Diego, CA). PCR primers were synthesized by Fudan Yueda Biotechnology Company (Shanghai, China). ELISA kits for measuring mouse IFN-γ, IL-17, IL-10 and TGF-β were purchased from eBioscience. RPMI 1640 and fetal calf serum (FCS) were obtained from Invitrogen Life Technologies. Intracellular Foxp3 staining was performed using the relevent Fix/Perm Buffer set (Biolegend or eBioscience) according to manufacturer's recommendations. The cell fluorescence was measured using LSR II (BD) and data were analyzed using Flowjo software (TreeStar).

### Isolation of CD4^+^CD25^+^ cells and CD4^+^CD25^-^Nrp1^+^T cells

Spleen and lymph node cells were obtained from B6 mice and prepared as single cell suspension. CD4^+^T cells were isolated by negative selection using the CD4^+^ T cell Isolation Kit (Miltenyi) according to the manufacturer's instructions. Then, CD25^+^ cells were isolated by using a CD4^+^CD25^+^ Regulatory T Cell Isolation Kit (Miltenyi) according to the manufacturer's instructions. The remaining CD25^−^ cells were incubated with a rabbit anti-mouse Nrp1 antibody (AbCam) for 20 min on ice, washed, then incubated with goat anti-rabbit IgG microbeads (Miltenyi) and selected for CD4^+^CD25^−^Nrp1^+^T cells according to the manufacturer's instructions, as described in by Solomon et al. [16]. Isolated cells were cultured in RPMI1640 supplemented with 10% FCS.

### in Vitro Suppression Assays

Proliferation assays were performed in triplicate in 96-well plates. Freshly isolated CD4^+^CD25^−^Nrp1^+^T cells (2×10^5^/well, C57BL/6) were co-cultured with irradiated BALB/c (donor) splenocytes in 5% CO_2_ at 37°C for 72 h. ^3^H-thymidine (1 µCi/well, Amersham Pharmacia Biotech, UK) was added to the culture for the final 18 h and Cell proliferation was measured by ^3^H-thymidine incorporation using a liquid scintillation counter (Wallac, Turku, Finland).

### Cardiac transplantation and histopathological examination

Donor hearts (BALB/c) were heterotopically (intra-abdominally) transplanted into recipient mice (C57BL/6). The aorta and pulmonary arteries of the donor hearts were end-to-side anastomosed to the recipient's abdominal aorta and inferior vena cava, respectively. Survival of cardiac allografts was evaluated by daily palpation; cessation of beating was interpreted as rejection. Recipient mice received a subtherapeutic regimen of 1 mg/kg/day i.p. Rapamycin (Sigma-Aldrich) in a vehicle containing 0.02% Tween 80 and 0.26% polyethylene glycol (both from Sigma-Aldrich) for 10 consecutive days (days 0–9), and/or two dose of freshly isolated CD4^+^CD25^−^Nrp1^+^T cell on day 0 and day 7.

The study endpoint was defined as complete cessation of cardiac beat. Survival of cardiac grafts was monitored by palpitation by two independent observers without prior knowledge of the treatment protocol, which was always confirmed with histology. Cardiac grafts were harvested whenever necessary, fixed in 10% formalin and embedded in paraffin. Sections were cut at 4 µm, and were counterstained for 1 min with hematoxylin and eosin.

### Quantitative real-time PCR (qRT-PCR)

Total tissue RNA was extracted using TRIzol (Invitrogen) reagent according to the manufacturer's instructions. cDNA was synthesized using oligo d(T) (Applied Biosystems) and a SuperScript III Reverse Transcriptase Kit (Invitrogen). A StepOne™ Real-Time PCR System (Applied Biosystems) and a SYBR RT-PCR kit (Takara) were used for quantitative real-time RT-PCR analysis. All reactions were conducted in a 20 ul reaction volume in triplicate. The relative expression levels for a target gene were normalized by *GAPDH*. Specificity of qRT-PCR was verified by melting curve analysis and agarose gel electrophoresis. Primer sequences used in qRT-PCR analysis are: TGF-β (5′-CCA CCT GCA AGA CCA TCG AC-3′; 5′-CTG GCG AGC CTT AGT TTG GAC-3′); IL-17A (5′-TCA GCG TGT CCA AAC ACT GAG-3′; 5′-CGC CAA GGG AGT TAA AGA CTT-3′); IL-10 (5′-GCT CTT ACT GAC TGG CAT GAG-3′; 5′-CGC AGC TCT AGG AGC ATG TG-3′); IFN-γ (5′-GAA CTG GCA AAA GGA TGG TGA-3′; 5′-TGT GGG TTG TTG ACC TCA AAC-3′); Foxp3 (5′-TCA AGT ACC ACA ATA TGC GAC C-3′; 5′-CCA TCG GAT AAG GGT GGC A-3′); Nrp1 (5′-ACC TCA CAT CTC CCG GTT ACC-3′; 5′-AAG GTG CAA TCT TCC CAC AGA-3′); and GAPDH (5′-TGA CCA CAG TCC ATG CCA TC-3′; 5′-GAC GGA CAC ATT GGG GGT AG-3′). Data were analyzed using the comparative C_t_ (2^−ΔΔCt^) method.

### Statistical analysis

Data from multiple groups were analyzed using one way ANOVA with post-hoc Bonferroni's correction (GraphPad Prism 5.0; GraphPad Software). Data derived from two groups were analyzed using an unpaired Student's t test or a Mann-Whitney test (two tailed). Survival rate was analyzed by the Kaplan-Meier method, and comparisons were made by log-rank analysis. All data were expressed as mean ± SD. In all cases, p<0.05 was considered with statistical significance.

## Results

### 1. CD4^+^CD25^−^Nrp1^+^ T cells possess potent suppressive function *in vitro*

**Figure 1:**
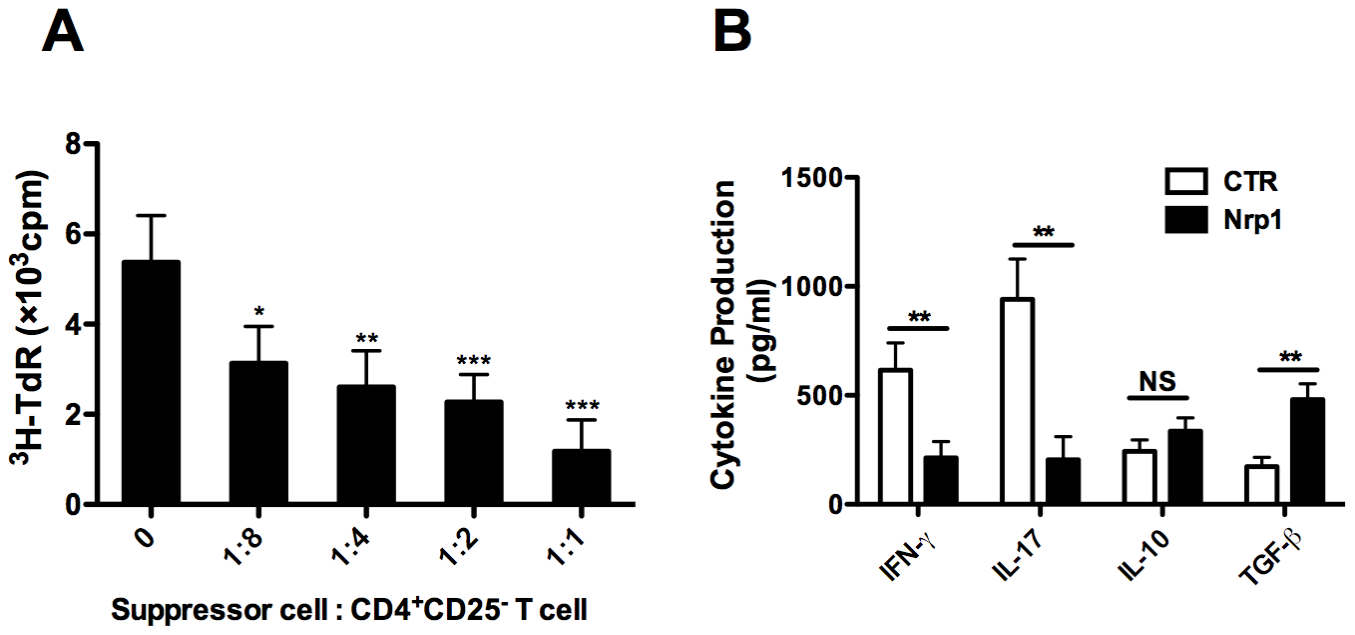
CD4^+^CD25^−^Nrp1^+^ T cells possess potent suppressive function *in vitro.* (**A**) Freshly isolated CD4^+^CD25^−^Nrp1^+^ T cells (10^5^, C57BL/6) were co-cultured with syngeneic responder CD4^+^CD25^−^ T cells (C57BL/6) in different ratios (0, 1∶8, 1∶4, 1∶2, 1∶1) in order to address stimulation induced by irradiated BALB/c (donor) splenocytes (10^5^). Cell proliferation was determined by ^3^H thymidine incorporation. (**B**) Cytokine content of the MLRsup where the suppressor cell versus CD4^+^CD25^−^ T cell was 0 (CTR) and 1∶1 (Nrp1) was evaluated by ELISA. Results are presented as mean ± SD values of triplicate wells, and are representative of 3 independent experiments. *P<0.05, **P<0.01, ***P<0.001. Nrp1 = neuropilin-1, MLRsup = mixed-lymphocyte reaction supernatants, CTR  =  control group, ^3^H-TdR  =  metabolic incorporation of tritiated thymidine, cpm  =  cells per million, NS  =  not significant.

We first addressed the *in vitro* suppressive function of freshly isolated CD4^+^CD25^−^Nrp1^+^ T cells by a standard inhibition assay. Freshly isolated CD4^+^CD25^−^Nrp1^+^ T cells in different ratios to responder CD4^+^CD25^−^ T cells were used to measure the inhibition of syngeneic CD4^+^CD25^−^ cell proliferation primed by irradiated BALB/c (donor) splenocytes. The results showed that CD4^+^CD25^−^Nrp1^+^ T cells were able to suppress the proliferation of CD4^+^CD25^−^ T cells, starting at 1∶8 ratios and showing 50% inhibition (IC50s) at 1∶ 4 ratios (Fig. 1A). We then quantified the cytokine content of the MLRsup by ELISA. At 1∶1 ratio to responder CD4^+^CD25^−^ T cells, CD4^+^CD25^−^Nrp1^+^ T cells suppressed the cytokine production of IFN-γ and IL-17, while increased the content of TGF-β as compared with the control group. Unexpectedly, no statistical difference was detected concerning the expression of IL-10 between CD4^+^CD25^−^Nrp1^+^ T cells treated group and the control group (Fig. 1B).

### 2. Adoptive transfer of CD4^+^CD25^−^Nrp1^+^ T cells synergize with Rapamycin to prevent allograft rejection

**Figure 2:**
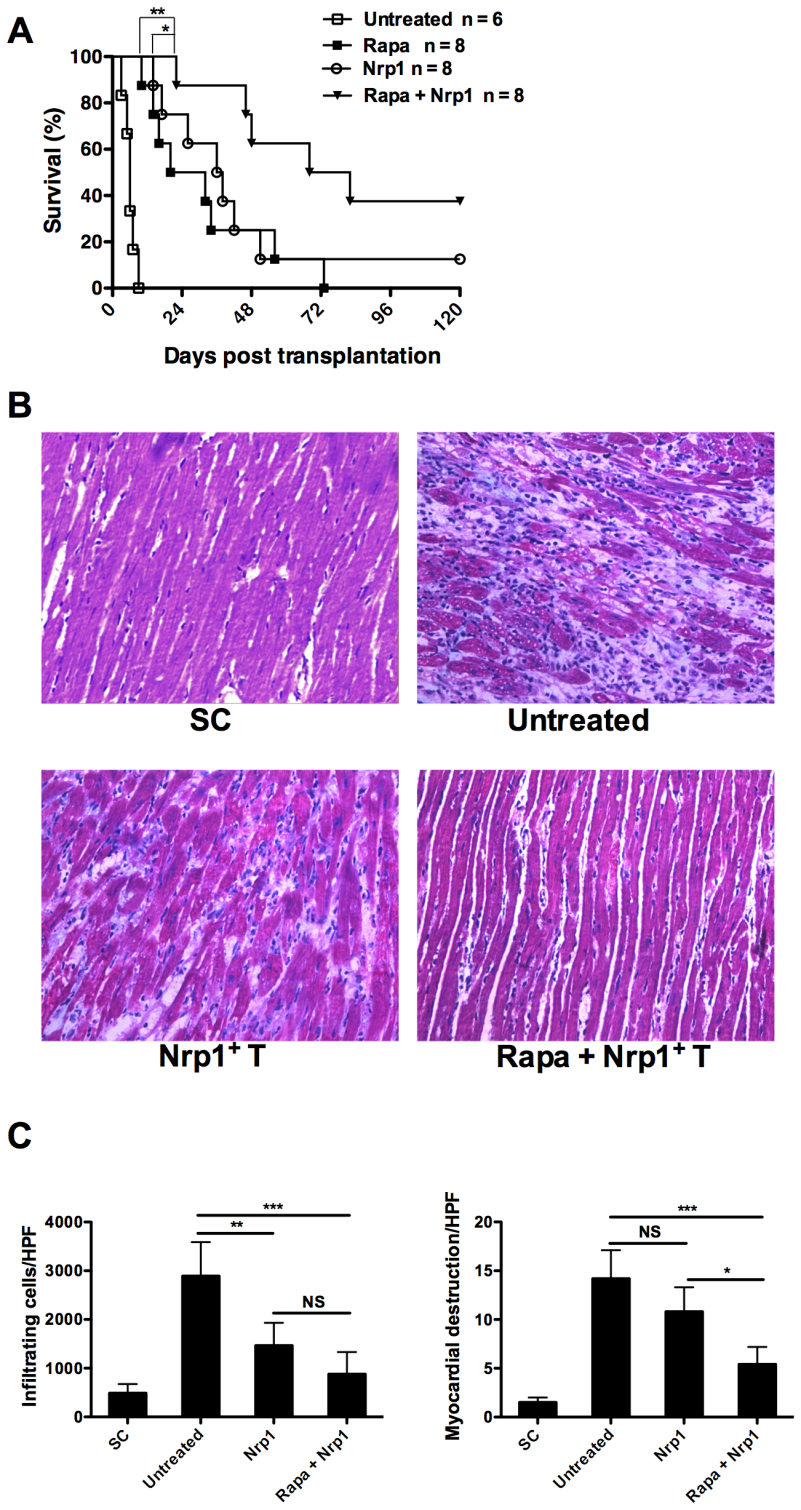
Adoptive transfer of CD4^+^CD25^−^Nrp1^+^ T cells synergize with Rapamycin to prevent allograft rejection. Heterotopic heart grafts were transplanted from BALB/c mice into C57BL/6 recipients. The recipients received a sub-therapeutic regimen of 1 mg/kg/day i.p. Rapamycin for 10 consecutive days (days 0-9), and/or two dose of freshly isolated Nrp1^+^ T cell on day 0 and day 7 (2×10^6^). Rejection was defined as cessation of a palpable impulse. (**A**) Survival rates were compared using log-rank test. (**B**) Hematoxylin and eosin staining of representative heart allografts harvested at 7d post transplantation. (**C**) Quantitative histological evaluation of allografts harvested on 7d post transplantation. SC, syngeneic control, Nrp1^+^ T = neuropilin-1-positive T cells, HPF = high power field, rapa = Rapamycin, NS = not significant. Results are presented as mean ± SD. *P<0.05, **P<0.01, ***P<0.001.

Next we sought to address the *in vivo* impact of CD4^+^CD25^−^Nrp1^+^ T cells on allograft rejection through a fully MHC-mismatched (BALB/cC57BL/6) murine abdominal heterotopic cardiac transplant model. Transplantation of syngeneic grafts (C57BL/6C57BL/6) served as controls. As shown in Fig. 2A, cardiac arrest occurred within one week if no treatment was given. Rapamycin or CD4^+^CD25^−^Nrp1^+^ T cells alone prolonged the median survival time (MST) to 26 days and 37 days, respectively. Combined therapy of CD4^+^CD25^−^Nrp1^+^ T cells and Rapamycin significantly prolonged the MST of cardiac allografts to 75 days, indicating that CD4^+^CD25^−^Nrp1^+^ T cells synergized with Rapamycin to prevent allograft rejection.

To confirm the above results, allografts from each study group were harvested on day 7 post-transplantation and subjected to histological analysis. While grafts from syngeneic transplantation had intact myocardial structure, the most severe inflammatory cell infiltration and destruction of myocardial tissue structure was present in untreated allografts (Fig. 2B). Importantly, although administration of CD4^+^CD25^−^Nrp1^+^ T cells significantly suppressed inflammatory infiltration, we still observed impaired myocardial structure in the allografts. On the contrary, administration of CD4^+^CD25^−^Nrp1^+^ T cells along with Rapamycin further reduced the damage to myocardial structure without perceptible changes in inflammatory infiltration (Fig. 2C, 2D). All these data support that CD4^+^CD25^−^Nrp1^+^ T cells synergized with a non-therapeutic dose of Rapamycin to prolong the survival of fully MHC-mismatched cardiac allograft.

### 3. Adoptive transfer of CD4^+^CD25^−^Nrp1^+^ T cells changes the intragraft and systemic inflammatory cytokine expression

**Figure 3:**
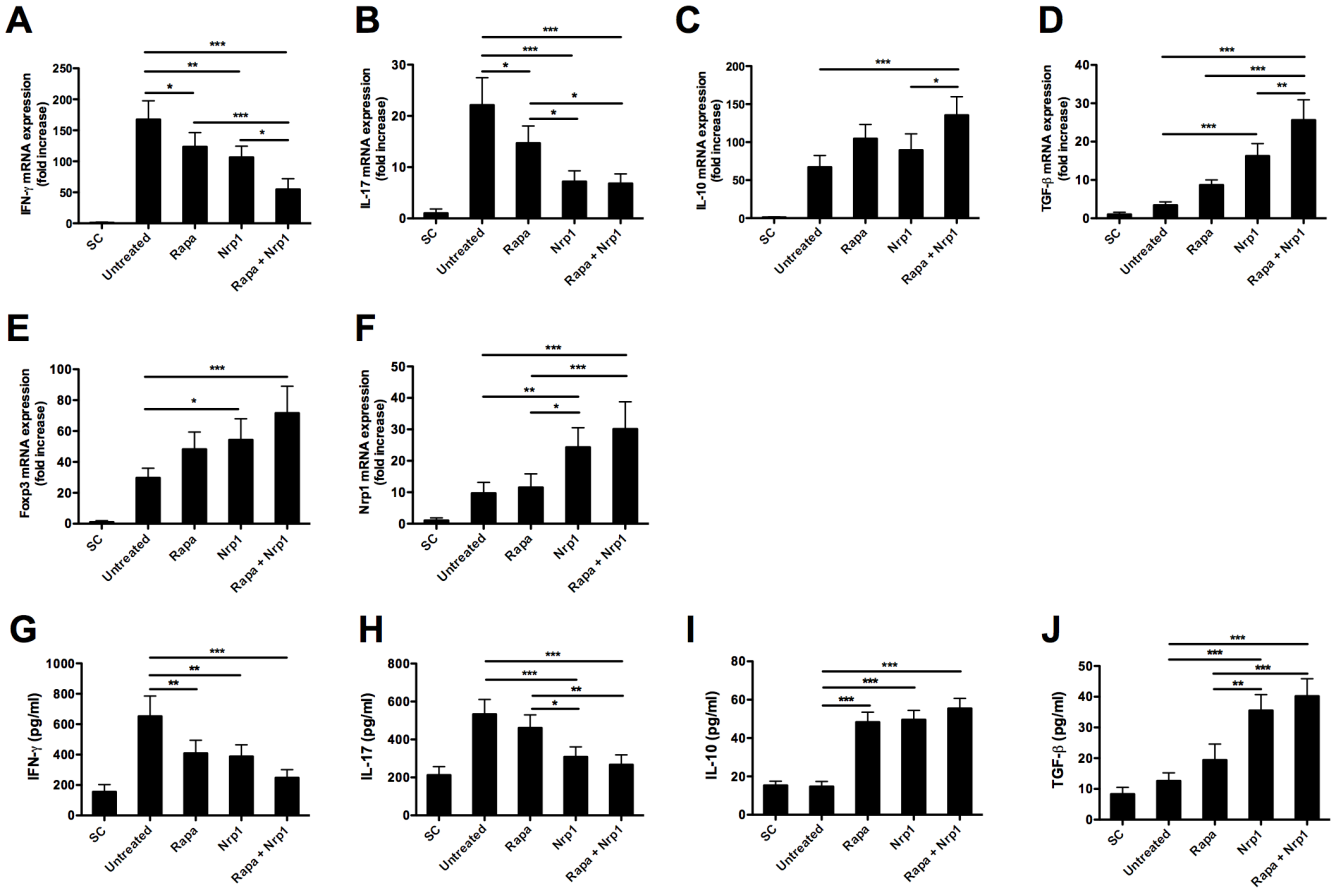
Adoptive transfer of CD4^+^CD25^−^Nrp1^+^ T cells changes the expression of inflammatory cytokines and immunomolecules.Both the cardiac allografts and blood samples were harvested 7 days after transplantation. (**A–F**) The intragraft gene expression of IFN-γ, IL-17, IL-10, TGF-β, Foxp3 and Nrp1 was analyzed by quantitative real-time polymerase chain reaction. (**G–J**) Serum levels of IFN-γ, IL-17, IL-10, TGF-β were determined by enzyme-linked immunosorbent assay. Results are presented as mean ± standard deviation. *P<0.05, **P<0.01, ***P<0.001. SC = syngeneic control, Nrp1 = neuropilin-1, rapa = Rapamycin.

Next, we examined the impact of CD4^+^CD25^−^Nrp1^+^ T cells on the expression of intragraft and serum inflammatory cytokines. To this end, on day 7 after transplantation, cardiac allografts were harvested for qRT-PCR analysis and blood was harvested for ELISA assay. Compared with allografts derived from untreated recipient mice, allografts from both Rapamycin and CD4^+^CD25^−^Nrp1^+^ T cells treated recipients showed significantly lower levels of IFN-γ and IL-17 expression, and combined therapy of Rapamycin and CD4^+^CD25^−^Nrp1^+^ T cells further reduced the intragraft expression of IFN-γ and IL-17 (Fig. 3A, 3B). In contrast, administration of Rapamycin together with CD4^+^CD25^−^Nrp1^+^ T cells significantly increased the intragraft expression of IL-10, while no discernable difference for expressions were detected in Rapamycin or CD4^+^CD25^−^Nrp1^+^ T cells alone treated mice in comparison with untreated control (Fig. 3C). Meanwhile, administration of CD4^+^CD25^−^Nrp1^+^ T cells rather than Rapamycin significantly increased the intragraft expression of TGF-β, and combined therapy of Rapamycin and CD4^+^CD25^−^Nrp1^+^ T cells further increased TGF-β expression (Fig. 3D). We also detected increased expression of Foxp3 and Nrp1 mRNA in the CD4^+^CD25^−^Nrp1^+^ T cells but not Rapamycin-only treated recipients. Foxp3 and Nrp1 mRNA levels further increased in the mice treated with the combination of both therapies as compared with the untreated controls. Even though the Rapamycin-only treated mice showed lower Nrp1 mRNA expression within the grafted tissues, almost similar levels of Foxp3 expression in comparison with the CD4^+^CD25^−^Nrp1^+^ T cells-only treated mice was observed (Fig. 3E, 3F). On the protein level, we also detected significantly decreased expression of IFN-γ and increased expression of IL-10 in the serum of mice treated by Rapamycin, CD4^+^CD25^−^Nrp1^+^ T cells alone or together treated mice as compared with that in untreated recipient mice (Fig. 3G, 3I). In addition, CD4^+^CD25^−^Nrp1^+^ T cells rather than Rapamycin decreased the expression of IL-17 and increased the expression of TGF-β in the serum (Fig. 3H, 3J). Together, we confirmed that CD4^+^CD25^−^Nrp1^+^ T treatment changed the intragraft and systemic expression of inflammatory cytokines towards an anti-inflammatory status.

### 4. CD4^+^CD25^−^Nrp1^+^ T cells augment CD4^+^Foxp3^+^ Treg accumulation in transplant recipients

**Figure 4:**
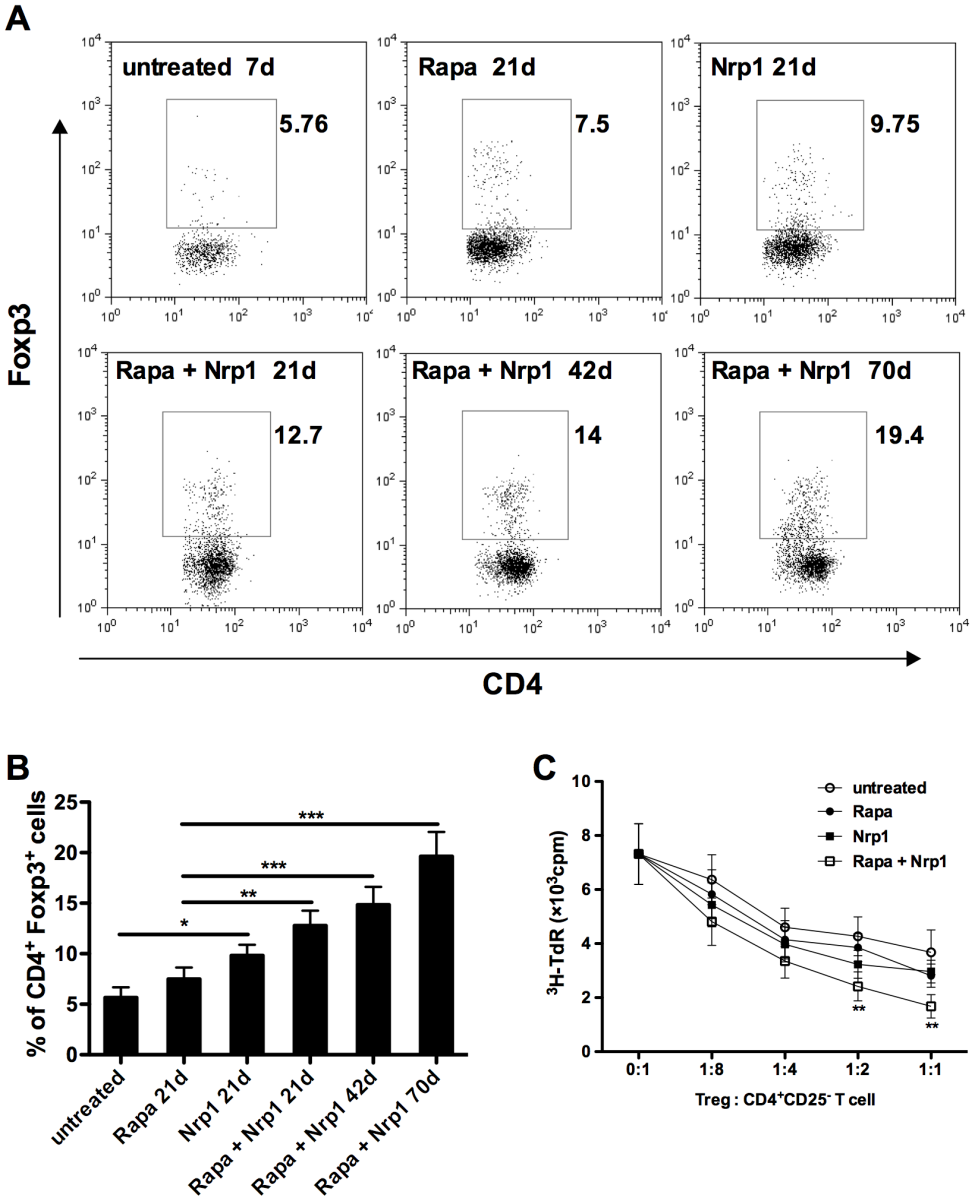
CD4^+^CD25^−^Nrp1^+^ T cells augment CD4^+^Foxp3^+^ Treg accumulation in transplant recipients. (**A**) Anti-CD4 and anti-Foxp3 intracellular staining were performed on spleen cells harvested from untreated mice on 7d or from Rapamycin and/or CD4^+^CD25^−^Nrp1^+^ T cells on 21d, 42d and 70d. (B) The percentages of CD4^+^Foxp3^+^ T cells were pooled from 4–6 mice from each group. (C) CD4^+^CD25^+^ T cells were purified from each group and used for suppression assays. 2×10^4^ CD4^+^CD25^−^ T cells (C57BL/6) were stimulated by the same amount of irradiated BALB/c splenocytes together with various doses of CD4^+^CD25^+^T cells purified from the indicated group. Cell proliferation was determined by ^3^H thymidine incorporation. Results are presented as mean ± SD values of triplicate wells, and are representative of 3 independent experiments. *P<0.05, **P<0.01, ***P<0.001. Rapa  =  Rapamycin, Nrp1 = neuropilin-1, ^3^H-TdR = metabolic incorporation of tritiated thymidine, cpm = cells per million, Treg = T regulatory cells

CD4^+^Foxp3^+^ Treg cells have been shown to be critically involved in the induction and maintenance of transplant tolerance in a broad range of studies. Following our observation that CD4^+^CD25^−^Nrp1^+^ T cells could prolong cardiac allograft survival, we tested whether CD4^+^Foxp3^+^ Treg cells could be involved in this mechanism. Indeed, on day 21 post-transplantation, we detected significantly increased CD4^+^Foxp3^+^ Treg cells in the spleens of CD4^+^CD25^−^Nrp1^+^ T cells but not Rapamycin-only treated mice as compared with untreated controls (P<0.05, Fig. 4A, 4B). Interestingly, the percentage of CD4^+^Foxp3^+^ Treg cells was further increased in mice that received combined therapy of CD4^+^CD25^−^Nrp1^+^ T cells and Rapamycin, and persistented in long-term allograft survivors that were sacrificed at day 42 and day 70 (Fig. 4A, 4B). Taken together, these data suggest that CD4^+^CD25^−^Nrp1^+^ cell transfer can augment CD4^+^Foxp3^+^ Treg accumulation in transplant recipients as a possible mechanism to prolong survival.

To determine whether these CD4^+^Foxp3^+^ Treg cells have a regulatory capacity, CD4^+^CD25^+^T cells were purified from spleens of mice sacrificed on day 21. By this method 76–83% of these CD4^+^CD25^+^T cells were determined to be Foxp3^+^, which were then used in a suppression assay to determine their function. As shown in Fig. 4C, better suppressive capability in a dose-dependent matter was found in CD4^+^CD25^+^ Treg cells purified from recipient mice treated by Rapamycin combined with CD4^+^CD25^−^Nrp1^+^ T cells as compared with those from untreated recipient mice.

### 5. CD4^+^CD25^−^Nrp1^+^ T cells induce hyporesponsiveness of the T effector cells

**Figure 5:**
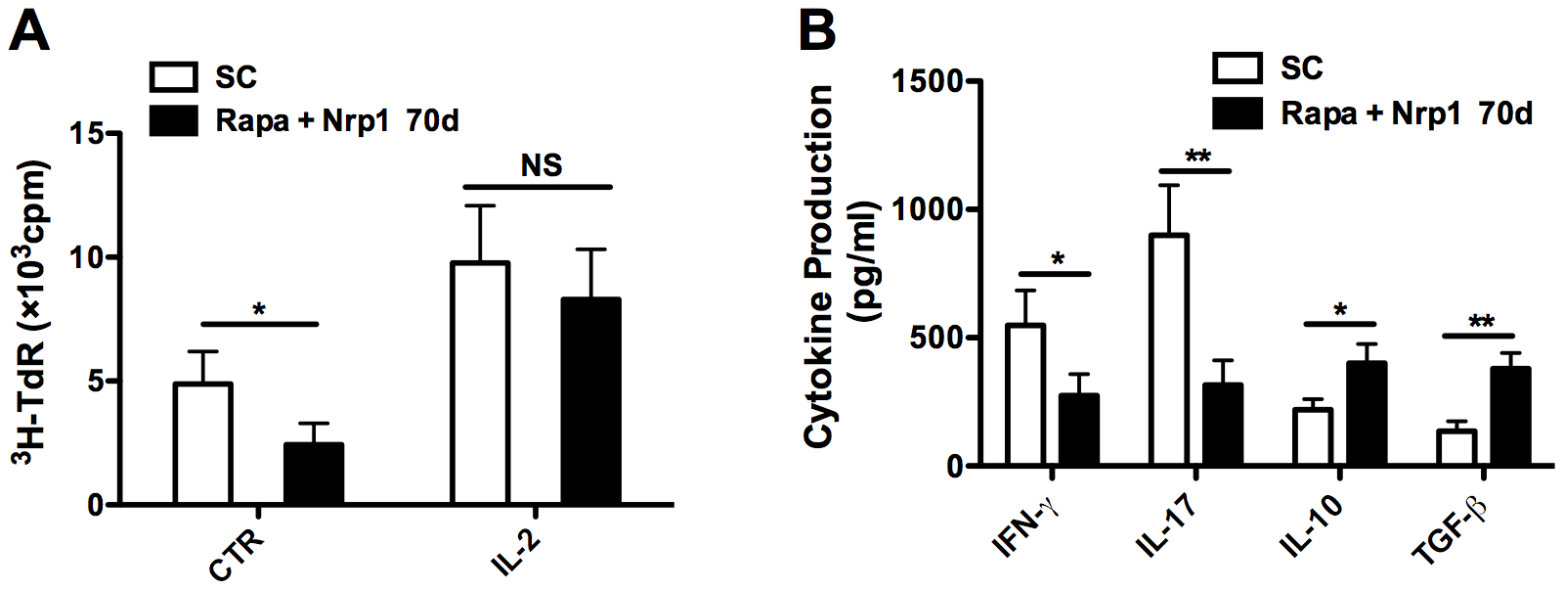
CD4^+^CD25^−^Nrp1^+^ T cells induce hyporesponsiveness of the T effector cells. CD4^+^CD25^−^ T cells were isolated from spleens of recipient mice treated by Rapamycin combined with CD4^+^CD25^−^Nrp1^+^ T cells and from syngeneic transplant recipients on day 70 after transplantation, then primed with irradiated BALB/c (donor) splenocytes in the presence or absence of exogenous IL-2 (100 U/ml). (**A**) Cell proliferation was determined by ^3^H thymidine incorporation. (**B**) Cytokine content of the mixed lymphocyte reaction supernatants with no exogenous IL-2 treatment was evaluated by enzyme-linked immunosorbent assay. Results are presented as mean ± SD values of triplicate wells, and are representative of 3 independent experiments. *P<0.05, **P<0.01, ***P<0.001. SC  =  syngeneic controls, ^3^H-TdR  =  metabolic incorporation of tritiated thymidine, cpm  =  cells per million, Nrp-1  =  neuropilin-1, rapa  =  Rapamycin, CTR  =  control, NS  =  not significant.

To further dissect the mechanisms underlying the protection of CD4^+^CD25^−^Nrp1^+^ T cells against allograft rejection, we further examined its impact on T effector cells. We isolated CD4^+^CD25^−^ T cells from the spleens of recipient mice treated with Rapamycin combined with CD4^+^CD25^−^Nrp1^+^ T cells on day 70 after transplantation, and examined their proliferation upon the priming by irradiated BALB/c (donor) splenocytes. Syngeneic cardiac transplant recipients that were sacrificed at the same time post transplantation served as controls. As demonstrated in Fig. 5A, Rapamycin combined with CD4^+^CD25^−^Nrp1^+^ T cell treated mice showed a significant reduction (2-fold on average) in T cell proliferation. Interestingly, addition of exogenous IL-2 to the assay with CD4^+^CD25^−^ T cell responders caused an almost complete restoration of responsiveness, with no significant difference between the groups. This suggests that Rapamycin combined with CD4^+^CD25^−^Nrp1^+^ T cells created conditions that favored induction of an anergic state in alloreactive T cells, which might contribute to the long-term allograft survival. The cytokine content of the MLRsup demonstrated significantly suppressed expression of IFN-γ and IL-17 in Rapamycin combined with CD4^+^CD25^−^Nrp1^+^ T cell treated mice, as well as increased production of IL-10 and TGF-β in comparison with the syngeneic control (Fig. 5B).

## Discussion

Experimental and preliminary clinical evidence has demonstrated that graft tolerance could be achieved and maintained by regulatory cell therapy, including tolerogenic DCs and regulatory T cells, which are commonly referred to as CD4^+^CD25^+^Foxp3^+^ T cells [17], [18], [19]. However, the heterogeneity of regulatory T cells has been widely accepted[20]. Even though much attention has been paid to CD4^+^CD25^+^Foxp3^+^ T cells, other non-Treg immunosuppressive T cells such as CD8^+^, NKT, and γδ-TCR cells have described to be required *in vivo* to achieve tolerance [8], [9], [21]. Additionally, TCRαβ^+^CD3^+^CD4^−^CD8^−^NK1.1^−^ (double negative, DN) T cells have been found to prevent graft rejection in a murine cardiac transplant model [10], [22]. Obviously, lack of insight into the phenotypes and function of non-Treg regulatory cells *in vivo* is halting the implementation of their therapeutic use in clinical transplantation. The reason to perform this study was therefore to assess the suppressive function of CD4^+^CD25^−^Nrp1^+^ T cells in the setting of transplantation. We found that CD4^+^CD25^−^Nrp1^+^ T cells suppress anti-donor T cell responses *in vitro*. In combination with short-term immunosuppression (non-therapeutic dose of Rapamycin), CD4^+^CD25^−^Nrp1^+^ T cells significantly prolonged the survival of heart grafts in a fully MHC-mismatched model.

Nrp1 was initially described as semaphorin-3A and VEGF receptor, being essential for axonal guidance and vascularization, respectively [11], [12]. In the immune system, Nrp1 has been shown to be involved in the priming of T cells by DC [23] and in the regulation of the immunological synapse and response [24], [25]. According to the study from Bruder and colleagues [13], Nrp1 represents a surface marker for the identification of Treg cells. Because it is highly expressed on CD4^+^CD25^+^ cells, and significantly down-regulated in activated CD4^+^CD25^–^ T cells. Moreover, CD4^+^Nrp1^high^ T cells suppress proliferation of naive CD4^+^CD25^–^ T cells, whereas CD4^+^Nrp1^low^ cells lack this capacity. However, as reported by Milpied *et al.*, Nrp1 might only represent a novel activation marker of human T cells but not a specific marker of human Tregs, because human Foxp3^+^ Tregs do not specifically express Nrp1 and Nrp1 expression is induced on peripheral blood T lymphocytes upon *in vitro* activation as well [26]. Inconsistently, another study has found a population of Nrp1^+^ Treg in human lymph nodes with Foxp3 expression that exerted contact-dependent suppression of T cell proliferation [27]. Recently, Nrp1 has been reported to be expressed at high levels by most natural Tregs, but at low levels by mucosa-generated and other noninflammatory inducible Tregs, which therefore makes Nrp1 a good surface marker to distinguish natural and inducible Tregs *in vivo* [28], [29]. While these contradictory results remain to be explained by further investigation, Battaglia's finding that Nrp1 was also expressed on some CD4^+^CD25^int^ and CD4^+^CD25^−^ T cells correlates well with our previous findings in mouse [15], [27].

It has been reported that Nrp1 expressed on Tregs prolongs the interaction between Tregs and immature DCs, allowing Tregs more time to recognize MHC class II-peptide complexes presented by iDCs, which results in higher sensitivity when limiting amounts of antigen are present[30]. Moreover, Nrp1 is a receptor for TGF-β1 and activates its latent form, which is also relevant to the Tregs' activity [31]. These findings suggest that besides being a surface marker, Nrp1 could be a key player in the list of molecules by which regulatory cells exert their suppressive effects. This raises the question whether Nrp1-expressing non-Tregs cells have suppressive function or not. Actually, in the context of autoimmune inflammatory responses, CD4^+^CD25^−^Nrp1^+^ T cells exhibited an as potent suppressive function as CD4^+^CD25^+^Nrp1^+^ T cells in preventing autoimmune encephalomyelitis [16]. Here in this study, we demonstrated strong suppressive function of CD4^+^CD25^−^Nrp1^+^ T cells both *in vitro* and *in vivo*, which correlated well with Solomon's findings in the EAE model. CD4^+^CD25^−^Nrp1^+^ T cells were reported to exert their suppressive function through TGF-β-dependent but not IL-10-dependent pathways during autoimmune inflammatory responses. Although we did not address this in our study, we did observe significantly elevated expression of both TGF-β and IL-10 in the CD4^+^CD25^−^Nrp1^+^ T cells treated mixed lymphocyte reaction supernatants, recipient mice sera and allograft homogenates.

Our results showed a decrease in IFN-γ and IL-17 cytokines in recipients that have received CD4^+^CD25^−^Nrp1^+^ T cells, indicating suppressed Th1 and Th17 response. IL-17 was reported to be a product of neutrophils during the early postoperative period and subsequently by Th17 and CD8^+^ T cells during allograft rejection in mice [32]. Consistently, we found that administration of CD4^+^CD25^−^Nrp1^+^ T cells significantly suppressed inflammatory infiltration in the allograft. Meanwhile, we also observed increased frequency of CD4^+^Foxp3^+^ T cells in the long-term surviving CD4^+^CD25^−^Nrp1^+^ T cells treated mice, suggesting an imbalance of Th17/Tregs towards the accumulation of Tregs. The increased expression of TGF-β might be one promoter for the development of CD4^+^Foxp3^+^ T cells [33]. However, the exact sequence of events that is induced by CD4^+^CD25^−^Nrp1^+^ T cells treatment during transplant immune response as well as their exact correlations remains to be investigated. Analysis of T cell reactivity in long-term surviving grafts from recipients indicated that, at 70 days post-transplant, the combination of Rapamycin and CD4^+^CD25^−^Nrp1^+^ T cells promotes conversion of alloreactive T cells to an anergic state, which seems to be another possible mechanism for the protection induced by CD4^+^CD25^−^Nrp1^+^ T cells against allograft rejection.

Rapamycin has been shown to be able to exert synergistic effects together with Tregs in preventing *in vivo* allorejection, including freshly isolated, *in vitro* or *in vivo* expanded, and antigen specific Tregs, while Tacrolimus and Cyclosporine A displayed opposite effects when combine used with Treg [7], [34], [35]. We found in this study that Rapamycin alone can suppress the pro-inflammatory and potentiates the anti-inflammatory cytokine expression both in the recipients sera and in the allograft homogenates. However, Rapamycin alone failed to increase the CD4^+^Foxp3^+^ T cells frequency in the recipient's spleen. To date, two studies have described the interaction between Nrp1 and the mTOR pathway. Bae and colleagues describe that autophagy, which was induced by administration of Rapamycin, associated with a reduction in the expression of Nrp1 on the surface of endothelial and carcinoma cells, which is somewhat counter-intuitive with a direct intracellular synergistic effect[36]. Whether Rapamycin via autophagy induces the breakdown of Nrp-1 in CD4^+^CD25^−^ T cells as well is not known. Manns *et al*. describe that dose-dependent Nrp1-receptor complex stimulation with semaphoring-3A in axons, via the stabilization of GSK3-β also had upstream effects on the mTOR pathway, which resulted in altered protein synthesis and degradation[37]. Rapamycin, independent from semaphoring-3A stimulation, further potentiated these processes *in vitro*. According to the report of Raimondi *et al*., the innate immune response after organ transplantation may convert T effector cells to a state refractory to Treg suppression, and inflammatory cytokines such as IL-6 might play a critical role in this process. Rapamycin treatment can alleviate the inflammatory response after organ transplantation, and hence increase the suppressive function of Tregs. Consistently, we also found longer survival in the combined therapy group as compared with either Rapamycin or CD4^+^CD25^−^Nrp1^+^ T cells-only treated group.

In conclusion, we demonstrated in this study that CD4^+^CD25^−^Nrp1^+^ T cells synergized with Rapamycin to induce long-term graft survival in fully MHC-mismatched murine heart transplantation. More importantly, our data indicated that augmenting the accumulation of CD4^+^Foxp3^+^ Treg cells and creating conditions that favored induction of an anergic state in alloreactive T cells might be one of the underlying mechanisms for CD4^+^CD25^−^Nrp1^+^ T cells to prevent allograft rejection. Although the exact molecular mechanism of CD4^+^CD25^−^Nrp1^+^ T cell-mediated suppressive function calls for future investigation, our findings indicated the possible therapeutic potential of CD4^+^CD25^−^Nrp1^+^ T cells in preventing allorejection. CD4^+^Nrp1^+^ T cells might therefore be used in bulk as a population of immunosuppressive cells with beneficial practical properties concerning *ex vivo* isolation as compared to Foxp3^+^ Tregs. These results also suggest that the development and interaction of different types of suppressive cells are required for controlling immune responses *in vivo*.
